# Spontaneous coronary artery dissection: a potentially devastating cardiovascular disease in young women

**DOI:** 10.1002/clc.23393

**Published:** 2020-05-22

**Authors:** Igor Vaz, Ashish Kumar, Bertrand Ebner

**Affiliations:** ^1^ Department of Medicine Jackson Memorial Hospital Miami Florida USA; ^2^ Department of Critical Care Medicine St John's Medical College Hospital Bengaluru India

To the Editor,

We read with interest the review article by Bullock‐Palmer et al entitled “Emerging misunderstood presentations of cardiovascular disease in young women.”^1^

Spontaneous coronary artery dissection (SCAD) is an uncommon medical condition with potentially devastating effects that affects primarily young women; a population considered not at risk for coronary artery disease. The optimal management for this condition is yet unknown, mainly due to the lack of robust evidence, as mentioned in the article.

The article approaches very well the current knowledge about the condition and the currently recommended therapy; it does mention the use of beta‐blockers as a therapy to prevent a recurrence, and we would like her to highlight the proposed pathophysiology involved, that is based on the idea of reducing shear stress, similar to what is used in acute aortic dissection.^2^ Beta‐blocker therapy is also important as secondary prophylaxis; one prospective study^3^ with 327 patients suggested that the use of beta‐blockers was associated with reduced incidence of recurrent SCAD.

We agree that further investigation, especially with randomized clinical trials, should be pursued to be a better understanding of the treatment options for this pathology. Two large studies are currently ongoing: a prospective cohort (NCT02188069) and a registry (NCT01429727) of angiographic images of those patients. Hopefully, these studies will allow a better understanding and guide future research for SCAD.
